# Manufacturing Dense Thick Films of Lunar Regolith Simulant EAC-1 at Room Temperature

**DOI:** 10.3390/ma12030487

**Published:** 2019-02-05

**Authors:** Philipp Nieke, Jaroslaw Kita, Marc Häming, Ralf Moos

**Affiliations:** 1 Department of Functional Materials, University of Bayreuth, 95440 Bayreuth, Germany; functional.materials@uni-bayreuth.de; 2 Airbus Defence and Space, Microgravity Payloads, 88090 Immenstaad, Germany; marc.haeming@airbus.com

**Keywords:** moon, in situ resource utilization (ISRU), regolith simulant, dense thick films, room temperature impact consolidation (RTIC), aerosol deposition method (ADM), gas kinetic spraying, vacuum deposition

## Abstract

The Aerosol Deposition (AD, also known as gas kinetic spraying or vacuum deposition) method is a rather novel coating process to produce dense thick films directly from dry ceramic (or metal) powders on a variety of substrates without any heat treatment. Because of the similarity of the up to now used powders and lunar regolith, it is imaginable to use AD systems for future in situ resource utilization missions on the Moon planned by several space agencies. To test the feasibility of such an endeavor, the processability of lunar mare simulant EAC-1 by the AD method has been examined in this study. Three regolith films with an area of 25 × 10 mm^2^, and thicknesses between 2.50 µm and 5.36 µm have been deposited on steel substrates using a standard AD setup. Deposited films have been investigated by Laser Scanning Microscopy (LSM) and Scanning Electron Microscopy (SEM). Moreover, the roughness and Vickers hardness of the deposited films and the underlying substrates have been measured. It has been shown that dense consolidated films of regolith simulant can be produced within minutes by AD. The deposited films show a higher roughness and, on average, a higher hardness than the steel substrates. Since on the Moon, naturally available regolith powders are abundant and very dry, and since the required process vacuum is available, AD appears to be a very promising method for producing dense coatings in future Moon exploration and utilization missions.

## 1. Introduction

Sustainable exploration and long-term utilization of the Moon is the next major step in space exploration expanding human presence further into the solar system. As announced by major space agencies and organizations, the Moon is going to be the destination of several manned and unmanned space missions in the foreseeable future [1,2,3,4]. Especially the establishment of a “Moon village” has been promoted recently by the European Space Agency (ESA) [5].

Within that scope, and for the exploration of more remote objects in our solar system (e.g., Mars or meteorites), the usage of locally sourced materials, known as in situ resource utilization (ISRU), becomes more and more inevitable for reasons of mass, costs, and risk [6,7,8]. For lunar missions, the sourced material of interest is lunar regolith, since it covers almost the complete surface of the Moon [9]. Objectives of ISRU are, amongst others, the production of propellant, construction materials, life consumables, and metals [6,7,8]. In the literature, many ISRU approaches are discussed, e.g., additive manufacturing [10,11,12], sintering [8,13], or chemical reduction [14] of lunar regolith to name a few. However, to the best of the authors’ knowledge, coating technologies that use regolith as a feedstock have hardly been considered so far. Therefore, the potential benefits of the Aerosol Deposition (AD) method to produce regolith films are presented in this paper. 

The AD method is a rather novel coating process. On earth, it provides the possibility to produce dense thick films directly from ceramic (or metal) powders on a variety of substrates without any heat treatment [15]. Many applications in the area of functional ceramics or surface protections have been reviewed [15]. A major part of the research and development of the AD technique has been conducted in East Asia, namely Japan and the Republic of Korea [15]. Especially, Akedo et al. laid the foundations for today’s understanding of the underlying mechanism of the AD method, which is commonly referred to as room temperature impact consolidation (RTIC) [16,17]. 

**Figure 1:**
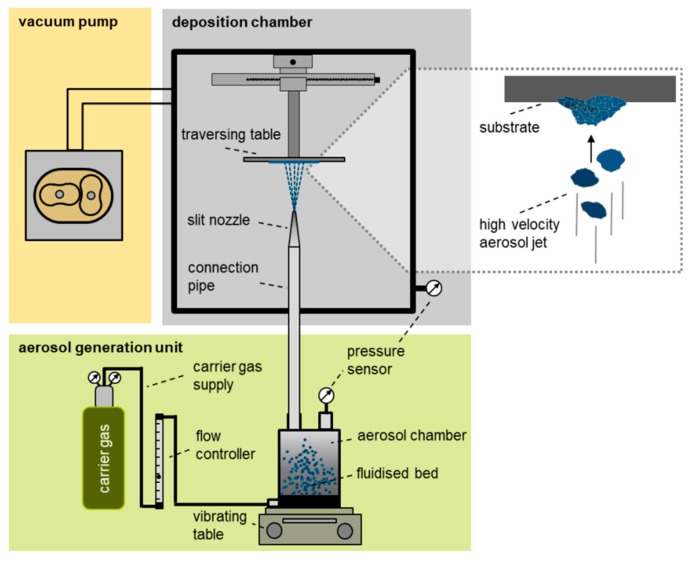
Scheme of aerosol deposition system at the Department of Functional Materials, University of Bayreuth, modified after Panzer et al. [18]. The inset shows impacting particles in the aerosol jet and already deposited particle fragments on the substrate.

A typical AD system is shown in Figure 1 and consists of three main parts: aerosol generation unit; deposition chamber; and vacuum pump. The aerosol generation unit is composed of a controlled carrier gas supply and an aerosol chamber. Inside the deposition chamber a slit nozzle and a traversing table with the substrate holder are located. The usual carrier gases are, e.g., oxygen, nitrogen, or helium. Oxygen may be most suitable in this context, due to its possible availability on ISRU missions regarding its potential use as propellant, in life supporting systems or as a product of the reduction of metal oxides [19]. The carrier gas flow is used to create an aerosol from the powder to be deposited (fluidized bed). Caused by the pressure difference between aerosol chamber and deposition chamber, the gas is accelerated in the nozzle to supersonic speed. Particles are accelerated by the drag force of the surrounding gas flow to velocities of several 100 m/s depending on their size, shape, and density. The high-speed aerosol jet impinges onto the steel substrate. Thereby, the solid particles impact on the substrate surface. This leads to break-up of particles to nanosized fragments, which in turn form the deposited dense film that consists of nanosized grains [15]. Subsequent impacting particles consolidate the deposited film by hammering and lead to film growth as well [15]. The entire process is therefore called room temperature impact consolidation (RTIC). Typically, a several micrometer thick coating can be produced within the order of minutes. In this way produced films characteristically feature nanograined microstructures, few and small pores, and therefore, high densities. Their optical, mechanical, and electrical properties often surpass bulk material properties. 

Owing to different system parameters, a comparable measure to quantify the AD process is the deposition rate r: it expresses the deposited film volume per unit time [15].

(1) $$r=v_{\mathrm{scan}}\cdot b_{\mathrm{film}}\cdot t_{\mathrm{film}}$$

In Equation (1), $v_{\mathrm{scan}}$ is the velocity of the traversing table, $b_{\mathrm{film}}$ is the width of the deposited film and $t_{\mathrm{film}}$ is the thickness of the film. 

On an ISRU mission, e.g., to the Moon, the abundantly available feedstock material for the AD method would be the lunar regolith. It forms the boundary layer between the space environment and the underlying solid lunar bedrock [9]. It consists of unconsolidated, fine-grained material that has been formed from solid lunar bedrock by random mechanism of the space environment. In younger lunar mare regions, a thickness of ~4–5 m, and in older lunar highland regions, a thickness of 5–15 m is expected [9]. Moreover, the chemical composition of the regolith varies significantly between these regions. Lunar regolith can be classified into different size fractions. Lunar soil is the term that describes the fraction <1 cm. Lunar soil has an average median of 70 µm and 10 to 20% of the soil is finer than 20 µm and therefore called lunar dust. Most often it is seen deleterious, especially to human respiratory tract [20] and because it causes engineering challenges and operational challenges [21]. Based on these figures, on average ~50% of the lunar soil would be possible feedstock for the AD process, especially the otherwise deleterious and often unutilized lunar dust. 

As a consequence of the limited availability of original lunar regolith, the use of simulant materials is a common practice to demonstrate new ISRU technology. EAC-1 is a lunar mare simulant that originates from the Eifel region near Cologne, Germany (see also Table 1) [8]. It has recently been published by the European Astronaut Centre.

Several up-to-now-used AD materials, mainly metal oxides resemble lunar soil or dust in their particle size distributions [15,22]. Examples for the successful application of the AD method for the processing of metal oxide powders include, e.g., Fe_2_O_3_ [23], SiO_2_ [24,25], TiO_2_, [26,27], and Al_2_O_3_ [16,28,29], or high temperature superconductors [30]. To the best of the authors’ knowledge, AD is the only process to produce dense ceramic coatings in the specified particle size range directly from dry powder and without any heat treatment. Therefore, it could be an enabler for future ISRU missions or a part of a process chain for other ISRU technologies that may require regolith films or coatings.

Moreover, the configuration of an AD unit on the Moon probably could be simpler and lighter than the terrestrial setup. A very basic setup would contain the aerosol generation unit including a pressurized carrier gas vessel. Substrates could be positioned for example in an open chamber that is maintained at low pressure due to a connection to the lunar vacuum environment or completely outside the AD unit. Therefore, the vacuum pump and possibly the vacuum chamber could become dispensable. Also, as a result of the reduced gravity on the moon [9], the aerosol generation might be facilitated whereby the vibrating table could become redundant. 

An important step to enhance the Technology Readiness Level (TRL) of AD systems is to test the processability of lunar dust simulant in laboratory environment. This is the objective of this paper.

## 2. Materials and Methods 

### 2.1. Material Preparation and Aerosol Deposition

The chemical composition of the EAC-1 is shown in Table 1 [8]. The received lunar regolith simulant EAC-1 was sieved with a 90 µm mesh and dried for >48 h at 200 °C in a drying oven to prevent powder agglomeration. Polished stainless steel sheets with a thickness of 1 mm were used as substrate material. Three substrates were coated on an area of 250 mm^2^. To ensure that the coating only takes place at the specified position the substrates were masked using conventional adhesive tape (Scotch Tape by 3M).

Deposition of the regolith simulant films was achieved using the AD system shown in Figure 1. The entire procedure took place at room temperature (about 25 °C). The most important system settings for the deposition are summarized in Table 2. 

### 2.2. Analysis

Particle size distributions of the received and the sieved EAC-1 material were measured using a Mastersizer 2000 (Malvern Instruments, Worcestershire, UK) equipped with Hydro 2000MU sample dispersion unit. As the refractive index of EAC-1 material was unknown, Fraunhofer approximation has been employed. 

The first film sample was investigated using the 3D Laser Scanning Microscope ZEISS LSM 800 Mat (Zeiss, Oberkochen, Germany), whereas the remaining two samples were examined for roughness, hardness, and film thickness. Roughness has been examined according to the German national standard DIN 4287 using a Waveline W20 profilometer (Jenoptik, Jena, Germany) equipped with TKL300 stylus. Scanning speeds of 0.5 and 0.15 mm/s were applied for deposited films and substrates, respectively. To determine the film thicknesses, the same device has been used. Film thicknesses have been measured at 8 positions per sample (4 at the masked top edge and 4 at the masked bottom edge, 5 mm distance between measurement positions). Microhardness was investigated via the Fischerscope H100 (Fischer, Sindelfingen, Germany) hardness measurement system. A force of 245 mN was applied for 10 s. Times for increase and decrease of the force were 10 s, respectively. Per sample, 24 indentations were made (12 at substrate and 12 at EAC-1 film). Moreover, cross-sections of the films were prepared by cutting the coated sample 3 using Accutom-50 (Struers, Willich, Germany). The cross-sections were embedded as well as grinded and polished using TegraPol-11 (Struers, Willich, Germany). Scanning electron microscopy (SEM) analyses of the cross-sections and EAC-1 powders were performed using a Zeiss Leo 1530 instrument (Zeiss, Oberkochen, Germany).

## 3. Results and Discussion

### 3.1. EAC-1 Powder Characterization

**Figure 2:**
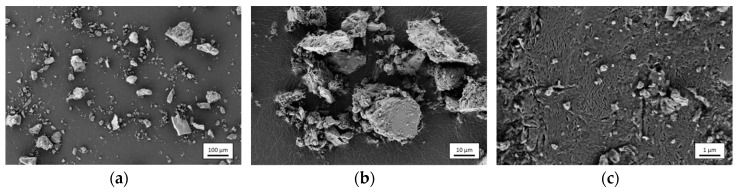
SEM images of as-received EAC-1 powder at (**a**) 100×, (**b**) 1000×, and (**c**) 10,000× magnification (SE detector).

**Figure 3:**
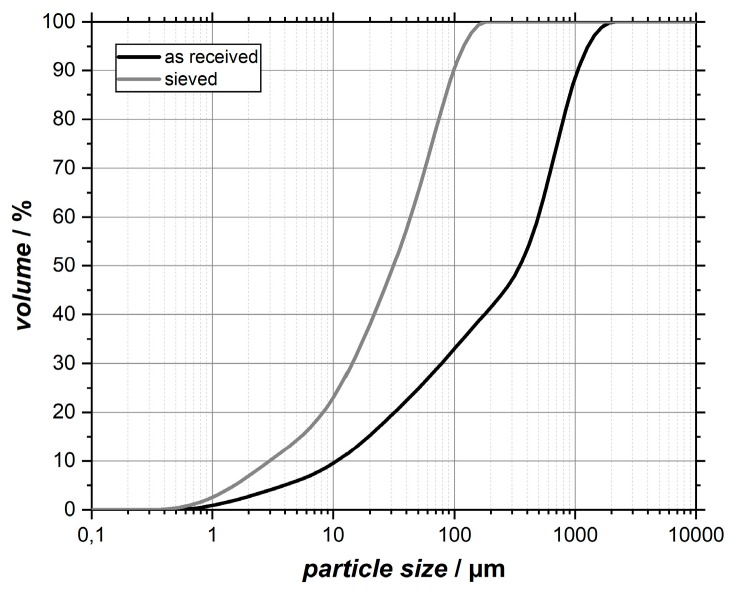
Cumulative particle size distributions of the as-received and the sieved EAC-1 material.

As described by Heiken et al. [9], particle shapes of original lunar soil particles show a large variety in their appearance. In Figure 2, SEM images of the as-received EAC-1 simulant powder are shown. A large variety in particle sizes, which ranges from >100 micron to nanoscale particles that are accumulated on larger grains (compare images (a) and (c) of Figure 2), can be found. Also, the particle morphology is mostly irregular and characterized by sharp edges. Through the subsequent sieving process, the largest particle fraction was removed. This is of importance as too big particles could negatively affect the film formation by abrasive blasting [15]. The achieved particle size distribution after sieving is visible in Figure 3 in comparison with the as-received material. It confirms that large particles actually were removed. Despite using a 90 µm mesh, a small amount of particles in the sieved powder seems to be larger than 90 µm. This observation could be attributed to a relative high aspect ratio of some grains that allow them to pass the mesh if right orientated (compare Figure 2). 

### 3.2. Deposited EAC-1 Films

**Figure 4:**
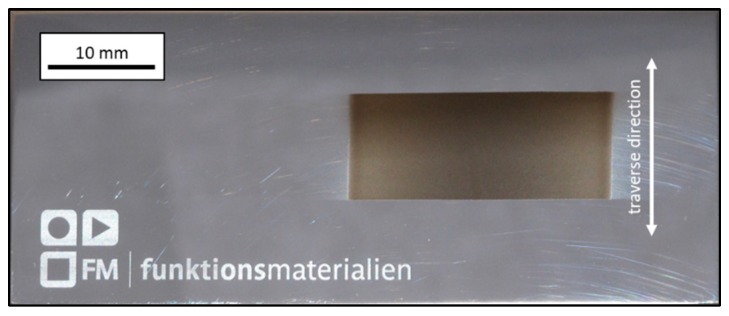
Image of 25 × 10 mm^2^ EAC-1 coating (dark grey) on a polished stainless steel substrate (70 × 30 mm^2^). The double arrow indicates the traverse direction of the substrate table. Film thickness approx. 3 µm, deposition time 2 min.

Application of the AD method led to successful deposition of EAC-1 regolith simulant on polished stainless steel substrates. Figure 4 exhibits an image of a coated substrate (sample 1), wherein the dark grey rectangular area is the actual EAC-1 film. The edges on top and bottom of the coated area are much sharper due to shadow masking with adhesive tape. On the left and on the right side no tape was used. It leads to a gradual transition from coating to substrate, which is sometimes referred to as overspray [31,32]. 

The processability of lunar regolith simulant to films in a laboratory environment is hereby shown. As composition on the lunar regolith varies significantly [9], AD should be tested on other simulants. If successful it may be possible to use AD on an ISRU mission in order to produce wear resistant (e.g., for excavation tools) or insulating coatings. In the following sections, some mechanical properties of the deposited films are examined. 

#### 3.2.1. Film Thicknesses and Deposition Rates at Steel 

The average film thicknesses of the manufactured coatings and their corresponding standard deviations are listed in Table 3. As could be expected from the system settings (see Table 2), the average film thicknesses of sample 1 and sample 2 are similar despite different measuring methods (LSM for sample 1 and profilometer for sample 2). Since the deposition time is directly proportional to the number of single scans, the deposition time for sample 3 is consequently twice as long as for the first two samples. For all three samples, a deposition rate of roughly 400 µm mm^2^/min has been achieved (calculated according to Equation (1)). Compared to some technical ceramic powders where deposition rates of 10,000 µm mm^2^/min were reported, this is a rather low value [15]. However, regolith simulant films of several µm in thickness can still be achieved within minutes and without any heat treatment. Moreover, this brief study with a non-optimized setup should only illustrate the feasibility to produce consolidated films of lunar regolith simulant by the AD method. An increase of deposition rate is probable by adjusting the process to the requirements for regolith (simulant). For instance, the particle size distribution of the feedstock could be selected more precisely. In addition, the tested setup is a transient batch process characterized by a non-constant particle concentration in the aerosol because of declining feedstock mass in the aerosol chamber. This means that less material is available for deposition in the last scans compared to the first scans. A constant feedstock supply would lead to constant particle concentration in the aerosol and therefore, most likely, to higher deposition rates. Finally, all other AD system settings mentioned in Table 2 could be adapted and offer a wide range for optimization. 

#### 3.2.2. LSM—Film and Substrate Surfaces

**Figure 5:**
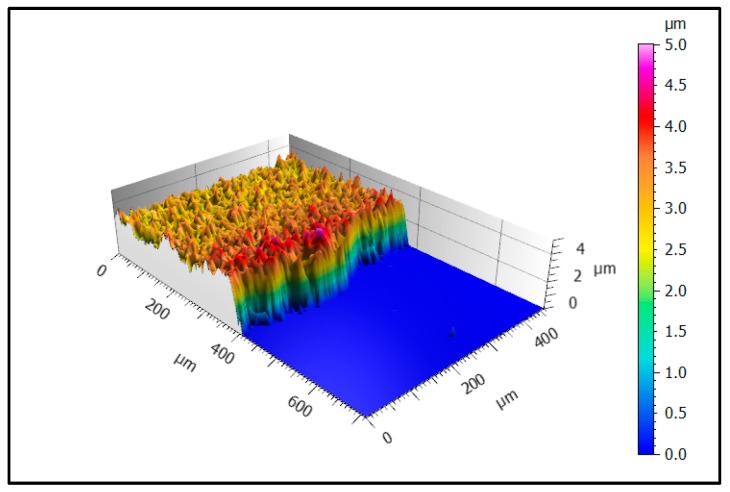
Pseudocolor image of the edge area of sample 1 showing the transition from substrate (blue) to deposited film (yellow/red) at 50× magnification.

Figure 5 shows a pseudocolor image of a typical part of the masked edge area of sample 1. The smooth blue area on the right hand side represents the steel substrate whereas the yellow/red surface illustrates the deposited EAC-1 film. In the investigated section, the thickness of the film is constant with a thickness ~3 µm, but the visible small thickness differences suggest clearly a higher roughness than the substrate. The slight thickness increase at the edge of the film may be attributed to the used shadow mask that could lead to higher deposition rates near the mask edge. 

#### 3.2.3. SEM—Cross-Section

**Figure 6:**
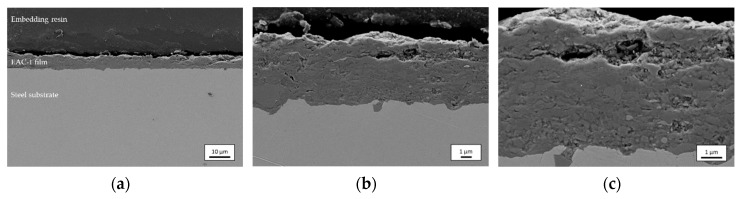
SEM cross-section of sample 3 at (**a**) 1000×, (**b**) 5000×, and (**c**) 10,000× magnification (SE detector). It was cut parallel to the traverse direction 10 mm from the right edge of the deposited film.

Figure 6 shows a cross-section of sample 3 at three magnifications: (a) 1000×, (b) 5000×, and (c) 10,000×. First of all, it becomes evident that the film thickness of the investigated cross-section corresponds to the tactile measured thickness of sample 3 (compare Table 3). Moreover, it is visible that a continuous connection between the substrate and the deposited film has been achieved. The bond is characterized by mechanical interlocking of the deposited particle fragments and roughness elements of the substrate that leads to good adhesion. This is a commonly observed feature of AD films [33,34]. However, another prepared cross-section shows small delamination effects in sections that may be caused by the shrinkage of the embedding resin during curing (not shown). At the highest magnification, the film microstructure becomes apparent. Particle fragments of sub-micrometer size are evident. They are thought to stem from impact processes at the substrate surface. Also, some small pores are visible in the intermediate space of the nanosized particle fragments. This is a typical behavior when irregularly shaped grains are stacked. Larger cavities at the surface of the film might be attributed to abrasive high-energy impacts of some large particles [15] or to the cutting process during the preparation of the sample. However, pores might negatively affect the mechanical properties of the deposited film and are therefore rather undesired in the most applications. Therefore, further work should focus on minimizing them. 

#### 3.2.4. Microhardness 

**Figure 7:**
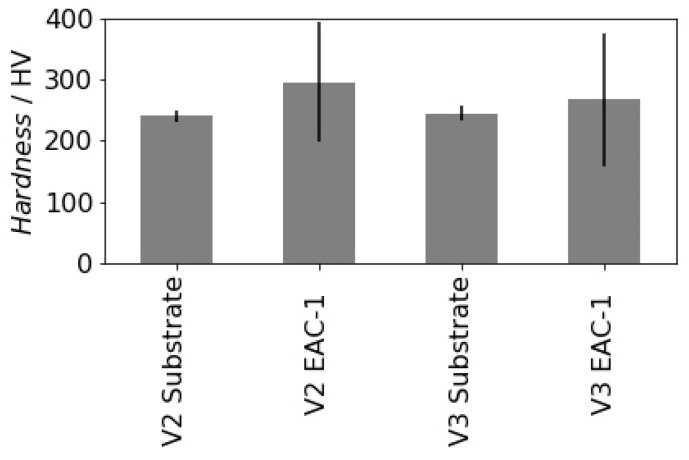
Average hardness values and corresponding standard deviations of substrates and deposited EAC-1 films.

To assess properties of the deposited film regarding future applications hardness measurements have been conducted. The measured averaged Vickers hardness (HV) values and the corresponding standard deviations are represented in Table 4 and illustrated in Figure 7. Steel substrates show typical mean hardness values and a small variation. Mean hardness values of the deposited films are 22.9% higher for sample 2 and 9.1% higher for sample 3 compared to the substrates hardness values. However, the standard deviation of the hardness increased considerably (around 10 times) compared to the reproducibly measured substrate hardness. This behavior is typical if one compares ductile metallic and brittle ceramic/glass materials. It may also be due to the naturally variable composition of the simulant material of softer and harder components. Another explanation might be the influence of pores, which reduce the effective cross-section of the film and therefore decrease their resistance to indentation. 

#### 3.2.5. Roughness 

Another crucial property of coatings is the surface roughness. A common measure for the roughness is the arithmetic average height *R*_a_ and the maximum height of the profile *R*_z_ [35] according to DIN 4287. Achieved roughness values of the two samples are high in comparison to the used substrates but can generally be classified as rather low (compare Table 5). While for many applications, a low roughness is desired, e.g., to minimize friction, for some applications a high roughness might be advantageous, e.g., for surface exchange processes. Therefore, it solely depends on the intended use of the deposited films in the ISRU process chain which characteristics should be enhanced in the future. 

## 4. Conclusions

It has been shown that the deposition of films comprised of regolith simulant EAC-1 on stainless substrates is possible without heat treatment using a standard aerosol deposition setup. Film thicknesses between 2.50 µm and 5.36 µm have been manufactured obtaining a deposition rate of ~400 µm mm^2^/min. Therefore, this work is seen as a step to enhance the TRL of the AD method for lunar applications. Morphology, hardness, and roughness of the deposited films have also been examined. These film properties might be improved through adaption of system parameters like nozzle geometry or a more precise selection of the feedstock material (e.g., by finer particle fractions). Further steps should include the development of an aerosol deposition unit to aerospace specifications (i.e., weight and volume reduction), as well as testing a complete adapted aerosol deposition unit in a vacuum environment and under low gravity conditions. One of the big advantages of the AD method is its relative independence of the chemistry of the feedstock material as chemical compositions of regolith at landing sites might be uncertain. A remaining requirement regarding the landing site is the availability of fine-grained material that could then be sieved to the appropriate particle size distribution for the AD process. If these conditions are met, the aerosol deposition could serve as a basic technology amongst others to produce lunar regolith films for scientific experiments or as a part of other ISRU methods. 

## Figures and Tables

**Table 1:** Chemical composition of EAC-1 as specified by the suppliers, after Schleppi et al. [8]. Listed oxides are not present in pure form but are incorporated in the mineral.

Oxides	Concentration in m.%
SiO_2_	43.7
Al_2_O_3_	12.6
Fe_2_O_3_	12.00
FeO	-
MgO	11.90
CaO	10.80
Na_2_O	2.90
K_2_O	1.30
TiO_2_	2.40
MnO	0.20
P_2_O_5_	0.60
Total	98.40

**Table 2:** Aerosol deposition (AD) system settings for the deposition of EAC-1.

Parameter	Sample 1	Sample 2	Sample 3
Mass of used simulant	21 g	20 g	44 g
Number of single scans	50	50	100
Substrate	Stainless steel sheets 70 mm × 30 mm × 1 mm		
Coated area	25 × 10 mm^2^		
Nozzle outlet width	25 mm		
Distance substrate – nozzle	2 mm		
Carrier gas	O_2_		
Volume flow	6 L/min		
Scan rate	5 mm/s		

**Table 3:** Overview of achieved film thicknesses, deposition times, and rates. Sample 1 was analyzed by laser scanning microscopy (LSM) and Samples 2 and 3 by profilometer, respectively.

Film and Deposition Properties	Sample 1	Sample 2	Sample 3
Mean film thickness ($t_{\mathrm{film}}$)/µm	2.91	2.50	5.36
Standard deviation µm	0.52	0.29	0.40
Deposition time/min	2	2	4
Deposition rate ^1^/(µm mm^2^/min)	436.5	375	402

^1^ Calculated according to Equation (1) and Table 2: $v_{\mathrm{scan}}$ = 5 mm/s, $b_{\mathrm{film}}$ = 25 mm (equals nozzle width).

**Table 4:** Average hardness values and corresponding standard deviations of substrates and deposited EAC-1 films.

Hardness	Sample 2		Sample 3	
	EAC-1	Substrate	EAC-1	Substrate
Mean/HV	295.33	240.26	267.45	245.07
Standard deviation/HV	97.75	8.39	108.53	12.37

**Table 5:** Roughness parameters *R*_a_ and *R*_z_ of the deposited EAC-1 films and substrates.

Roughness Parameters	Sample 2		Sample 3	
	EAC-1	Substrate	EAC-1	Substrate
*R*_a_/µm	0.36	0.01	0.44	0.01
*R*_z_/µm	2.83	0.08	3.25	0.08
